# Supplementary material for: Hybrid Silver-Containing Materials Based on Various Forms of Bacterial Cellulose: Synthesis, Structure, and Biological Activity
Source: Int J Mol Sci. 2023 Apr 21;24(8):7667. doi: 10.3390/ijms24087667 (PMC10142189; doi:10.3390/ijms24087667)
Supplement: Supplementary file 1 [file ijms-24-07667-s001.zip › ijms-2349710-supplementary.pdf]

Supplementary Materials

Hybrid Silver-Containing Materials Based on Various Forms of Bacterial Cellulose: Synthesis, Structure, and Biological Activity

Alexander Vasil'kov [1,*], Ivan Butenko [1,2], Alexander Naumkin [1], Anastasiia Voronova [1], Alexandre Golub [1], Mikhail Buzin [1], Eleonora Shtykova [3], Vladimir Volkov [3] and Vera Sadykova [2]

[1] A.N. Nesmeyanov Institute of Organoelement Compounds, RAS, 119334 Moscow, Russia
[2] G.F. Gause Institute of New Antibiotics, 119021 Moscow, Russia
[3] Shubnikov Institute of Crystallography, FSRC "Crystallography and Photonics" RAS, 119333 Moscow, Russia
* Correspondence: alexandervasilkov@yandex.ru

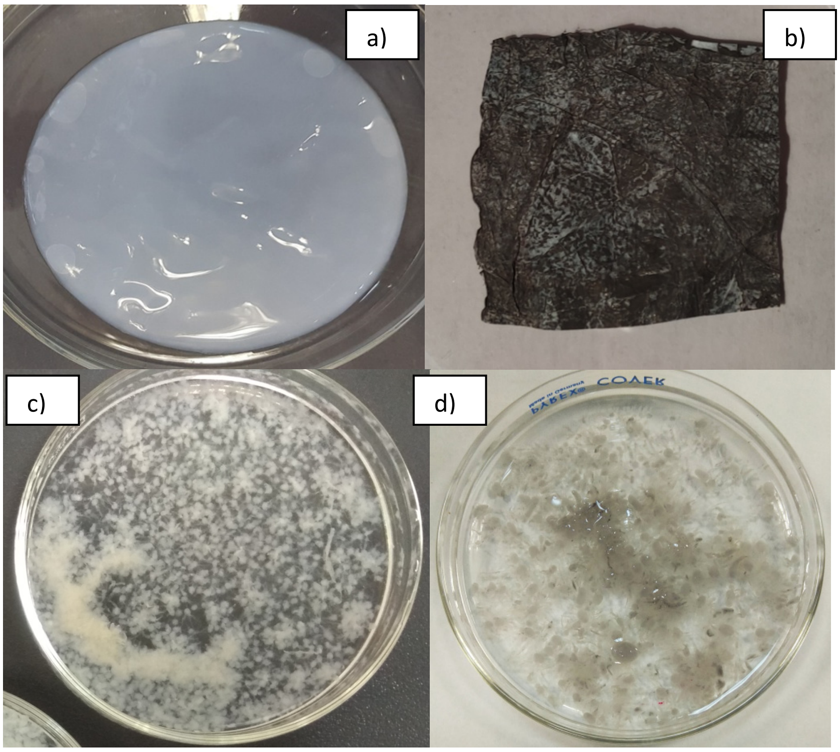

Figure S1. Initial and silver-containing bacterial cellulose in the form of films and spheres: BCF (a), Ag NPs/BCF (b), SBCB (c), Ag NPs/SBCB (d)

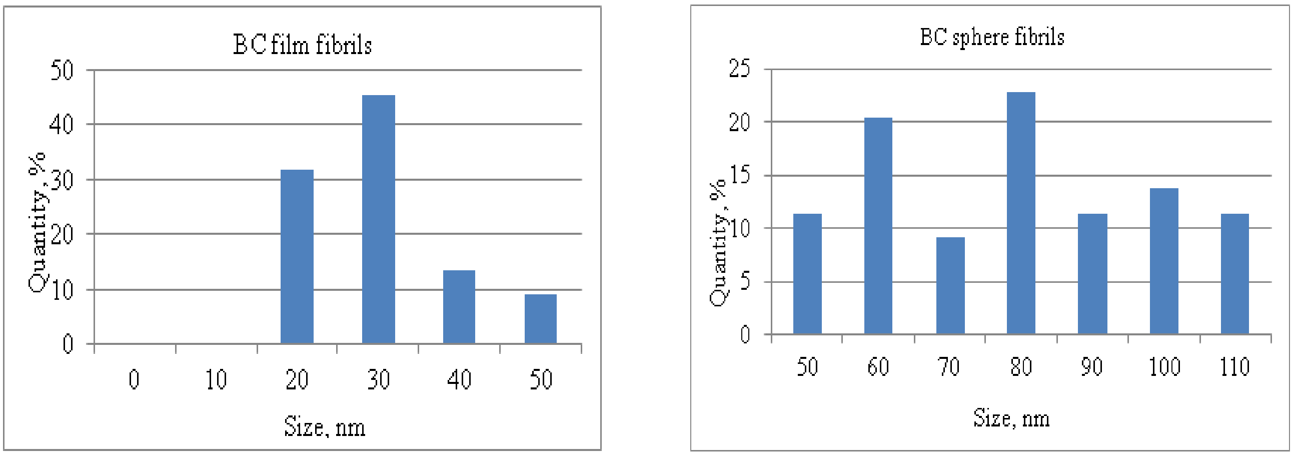

a b

Figure S2. Histogram of the thickness distribution of BCF (a) and SBCB (b) fibrils

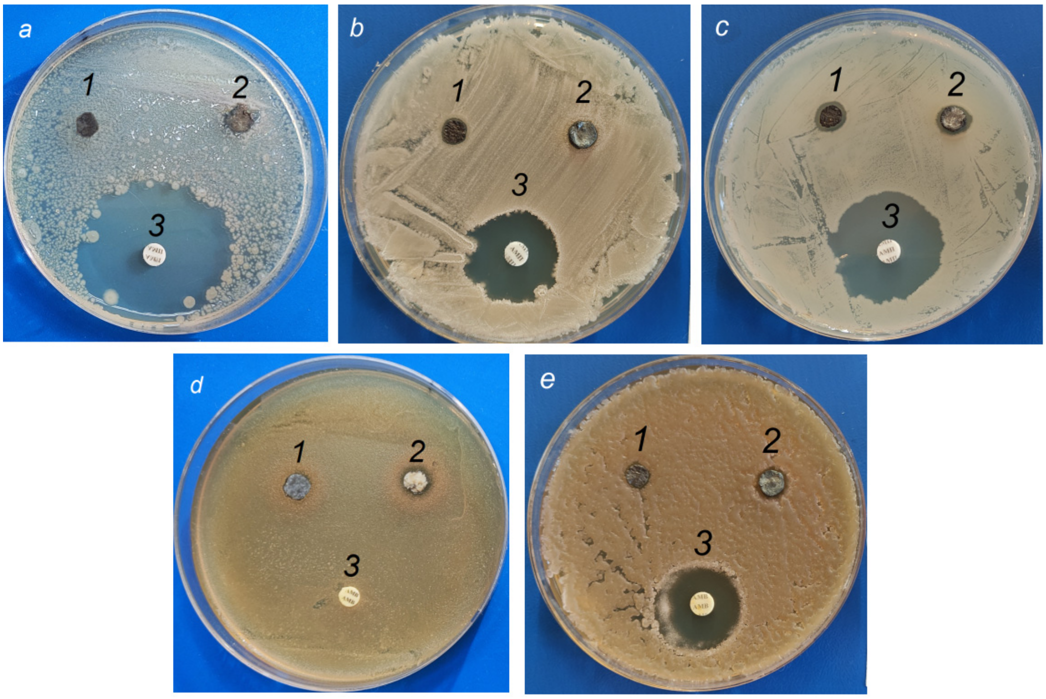

Figure S3. Antimicrobial activity of bacterial cellulose with Ag nanoparticles revealed by disk diffusion: 1 – Ag NPs/BCF; 2- Ag NPs/SBCB; 3 (for a, b and c) – Ampicillin, (for d and e) – Amphotericin B; where (a) - *B. subtilis* ATCC 6633; (b) – *S. aureus* ATCC 25923; (c) – *E. coli* ATCC 25922; (d) – *C. albicans* ATCC 2091; (e) – *A. niger* INA 00760
